# Primary Hepatic Neuroendocrine Neoplasms: Systematic Review and Expert Pathologist Reappraisal Using the WHO 6th Edition Framework

**DOI:** 10.1111/liv.70921

**Published:** 2026-10-13

**Authors:** Marcello Maestri, Ottavia Cicerone, Alessandro Gambella, Federica Grillo, Stefano La Rosa, Silvia Uccella, Alessandro Vanoli

**Affiliations:** ^1^ General Surgery Unit I—Liver Service Fondazione IRCCS Policlinico San Matteo Pavia Italy; ^2^ Department of Clinical‐Surgical, Diagnostic and Pediatric Sciences University of Pavia Pavia Italy; ^3^ Pathology Unit, Department of Surgical Sciences and Integrated Diagnostics (DISC) University of Genoa Genoa Italy; ^4^ Azienda Ospedaliera Metropolitana (AOM) IRCCS Ospedale Policlinico San Martino Genoa Italy; ^5^ Pathology Unit, Department of Medicine and Technological Innovation University of Insubria Varese Italy; ^6^ Department of Biomedical Sciences Humanitas University Milan Italy; ^7^ Department of Pathology IRCCS Humanitas Research Hospital Milan Italy; ^8^ Department of Molecular Medicine—Pathological Anatomy University of Pavia, Fondazione IRCCS Policlinico San Matteo Pavia Italy

## Abstract

Primary hepatic neuroendocrine neoplasms (PHNENs) are exceedingly rare. Published evidence often lacks adequate clinicopathological support for a primary hepatic origin and uses inconsistent historical terminology. Previous reviews have pooled well‐differentiated neuroendocrine tumours (NETs), poorly differentiated neuroendocrine carcinomas (NECs), and mixed neuroendocrine–non‐neuroendocrine neoplasms (MiNENs), despite differences in biology, prognosis, and management. We systematically reviewed reported PHNENs using PRISMA‐based screening and expert pathology reappraisal within the WHO 6th edition morphology‐first framework. A systematic literature search was conducted in PubMed/MEDLINE, Scopus, and Web of Science. Titles, abstracts, and full texts were screened according to predefined eligibility criteria; eligible articles underwent second‐level expert assessment of pathology, including morphology (including NET G3 vs. NEC and MiNEN vs. amphicrine carcinoma), immunohistochemistry (expression of at least two NE markers), proliferation (Ki‐67/mitotic activity), as well as clinical/imaging work‐up to exclude metastases from extrahepatic primaries. Of the 67 potentially eligible studies submitted to expert pathology‐based reappraisal, 28 studies (49.1%) were retained in the final dataset with 57 likely NEN cases (mainly case reports/small case series). Among the 57 cases, most were well‐differentiated NETs (78.9%); NECs (8.8%), MiNENs (3.5%), and amphicrine carcinomas (1.8%) were less frequent. Some cases were high‐grade NENs that could not be distinguished as well or poorly differentiated (7%). PHNEN literature is constrained by rarity, uncertainty of hepatic origin, and historical misclassification. Future studies and registries should report a minimum diagnostic dataset with standardised terminology according to the WHO 6th edition framework and in‐depth clinical/imaging studies.

## Key Points

- Primary hepatic neuroendocrine neoplasms are exceptionally rare and cannot be distinguished with certainty from liver metastases of an occult extrahepatic neuroendocrine neoplasm.
- Expert pathology adjudication retained only 28 of 67 potentially eligible studies after confirming compatible morphology, at least two recommended general neuroendocrine markers, and sufficient evidence excluding extra‐hepatic primary.
- Historical terminology frequently misuses well‐differentiated neuroendocrine tumours, poorly differentiated neuroendocrine carcinomas, and mixed neoplasms despite distinct biology and management.
- Among 57 classifiable cases, well‐differentiated neuroendocrine tumours predominated (78.9%); neuroendocrine carcinomas (8.8%), mixed neuroendocrine–non‐neuroendocrine neoplasms (3.5%), and amphicrine carcinomas (1.8%) were less frequent. Some cases were high‐grade neoplasms that could not be distinguished as well or poorly differentiated (7%).
- Hepatic origin remains a diagnosis of exclusion; standardised reporting and long‐term follow‐up with reassessment are therefore essential for diagnosis, multidisciplinary care, registries, and future research.

## Lay Summary

Neuroendocrine neoplasms that start in the liver are extremely rare and can be difficult to distinguish from neoplasms that have spread there from other organs. Our review of published cases found that many reports lacked enough information to support the diagnosis and that these neoplasms include distinct types that need different approaches to treatment. Expert examination of tumour tissue, thorough checks for neoplasm elsewhere in the body, and long‐term follow‐up are essential to guide appropriate care and build more reliable evidence for future patients.

## 1 Introduction

### 1.1 Background and Epidemiology

Primary hepatic neuroendocrine neoplasms (PHNENs) are among the rarest primary liver malignancies, comprising approximately 0.3% of all neuroendocrine neoplasms (NENs) and less than 0.5% of primary hepatic tumours [1, 2, 3]. First described by Edmondson in 1958, these tumours arise in a site not traditionally associated with a resident neuroendocrine cell population [4]. Because the liver is a frequent metastatic target of NENs, the discovery of a neuroendocrine lesion within the liver overwhelmingly suggests metastatic disease, and the diagnosis of a primary hepatic origin requires rigorous exclusion of extrahepatic primary sites [5, 6].

The extreme rarity of PHNENs has two consequences: (1) standardised treatment guidelines do not exist; therefore, management is extrapolated from protocols designed for metastatic gastroenteropancreatic NENs or hepatocellular carcinoma (HCC); and (2) published reviews have historically analysed all PHNEN histologic types as a single entity, conflating biologically distinct subgroups with fundamentally different prognoses [7, 8].

### 1.2 The Classification Imperative: Confirmation of Neuroendocrine Differentiation and Separation of NET, NEC, MiNEN, and Amphicrine Carcinoma

The WHO 6th edition requires morphological evidence of NE differentiation as the first diagnostic prerequisite and immunohistochemical (IHC) confirmation as the second. Because no single general NE marker is completely sensitive and specific, definitive confirmation requires positivity for at least two of chromogranin A, synaptophysin, and INSM1. CD56 expression alone is insufficient as it lacks specificity. Neuroendocrine differentiation must be determined as it is now firmly established in NE pathology that well‐differentiated neuroendocrine tumours (NETs) and poorly differentiated neuroendocrine carcinomas (NECs) are distinct disease entities rather than a morphologic continuum [9, 10, 11]. The critical distinctions are as follows:

- NETs are well‐differentiated neoplasms with organoid architecture, abundant granular cytoplasm, bland nuclei with ‘salt‐and‐pepper’ chromatin and small nucleoli, and strong expression of Synaptophysin and Chromogranin A. NET grade is assigned according to mitotic rate and Ki‐67 proliferation index, using the higher of the two parameters: G1, < 2 mitoses per 2 mm^2^ and Ki‐67 < 3%; G2, 2–20 mitoses per 2 mm^2^ and/or Ki‐67 3%–20%; and G3, > 20 mitoses per 2 mm^2^ and/or Ki‐67 > 20%. Molecular profiles are dominated by alterations in the MEN1 gene and chromatin remodelling pathways [9, 10].
- NECs are poorly differentiated carcinomas of small‐cell or large‐cell type, characterised by marked cytological atypia, high mitotic activity, geographic necrosis, and sheet‐like or diffuse growth. Poorly differentiated morphology, rather than a specific Ki‐67 threshold, defines NEC, although the Ki‐67 index is invariably high. Alterations involving TP53 and RB1 genes are frequent but are supportive rather than defining diagnostic features [9, 10, 11]. In the WHO 2026 classification of digestive system tumours, the presence or absence of tumour necrosis should be documented together with the grade in NETs, whereas this is not a mandatory reporting requirement for NECs [12].
- Mixed neuroendocrine‐non‐neuroendocrine neoplasms (MiNENs) contain spatially and morphologically recognisable and immunophenotypically confirmed NE and non‐neuroendocrine components (e.g., HCC or cholangiocarcinoma), each representing at least 30% of the tumour mass [13, 14]. By contrast, amphicrine carcinoma consists of a single population of tumour cells showing simultaneous NE and non‐neuroendocrine differentiation rather than two separate components. This concept can represent either individual tumour cells showing concurrent morphological and immunohistochemical evidence of NE and non‐neuroendocrine differentiation, or a morphologically uniform neoplastic population comprising distinct cell subsets that express neuroendocrine or non‐neuroendocrine markers, with an additional subset expressing both phenotypes in some cases. This distinction is now explicit in the WHO 6th edition and may be relevant in the liver, where hepatocellular and neuroendocrine differentiation may coexist within the same cells [13, 14, 15, 16, 17, 18, 19, 20, 21, 22, 23, 24, 25].

### 1.3 The WHO Classification Timeline and Its Impact on the Literature

The evolution of the WHO classification and its implications for interpreting the PHNEN literature are summarised in Table 1.

**TABLE 1:** Evolution of the WHO classification of neuroendocrine neoplasms and its implications for the interpretation of PHNENs.

WHO	Key change	Impact on PHNENs	References
Gastrointestinal Tumours, 4th Edition	A Ki‐67 index above 20% generally resulted in classification as NEC, irrespective of tumour differentiation.	This approach could misclassify well‐differentiated NET G3 as poorly differentiated NEC.	[15]
Tumours of Endocrine Organs, 4th Edition	NET G3 was introduced for well‐differentiated pancreatic tumours with proliferation above the G2 range.	Older high‐proliferation, well‐differentiated pancreatic tumours can be reinterpreted as NET G3 rather than NEC when supported by morphology.	[16]
Digestive System Tumours, 5th Edition	NET G3 was extended across the digestive system, and differentiation/morphology became the primary criterion separating NET from NEC.	Historical hepatic NEN labels can be reinterpreted using a morphology‐first framework.	[9]
Endocrine and Neuroendocrine Tumours, 5th Edition	The morphology‐first approach was confirmed; NEC commonly shows Ki‐67 above 55%, whereas NET G3 has no defining upper Ki‐67 limit.	Proliferation supports grading but does not replace differentiation in the NET G3 versus NEC distinction.	[10]
Digestive System Tumours, 6th Edition	Morphology‐first classification was retained. Neuroendocrine differentiation requires compatible morphology and positivity for at least two of chromogranin A, synaptophysin, and INSM1. Pure and non‐pure neoplasms are separated; amphicrine‐like carcinoma is distinguished from MiNEN; tumour necrosis is recorded in NET; and NEC is high grade by definition.	The reappraisal must verify the two‐marker requirement, distinguish NET G3 from NEC, and separate MiNEN from amphicrine‐like carcinoma.	[12]

The interpretation of the PHNEN literature is complicated by changes in WHO classification over time. Older studies frequently used terms such as ‘carcinoid tumour,’ ‘well‐differentiated neuroendocrine carcinoma,’ or ‘neuroendocrine carcinoma’ in ways that do not map directly onto current WHO categories. Under older classification systems, tumours with Ki‐67 above 20% were often categorized as NEC regardless of morphology. The current WHO morphology‐first framework classifies a well‐differentiated tumour with high proliferation as NET G3, whereas NEC requires poorly differentiated morphology. Abnormal p53 or loss of Rb expression and molecular alterations involving *TP53*, *RB1*, or related pathways may support NEC in difficult cases, but they do not replace morphology and are not mandatory for classification.

Therefore, excluding all older reports would discard potentially valuable cases, but accepting historical diagnostic labels without reassessment would introduce substantial classification bias. The present review was designed to address this problem by combining systematic literature screening with expert pathology adjudication [15, 16].

### 1.4 Rationale for Expert Pathology Adjudication

The central premise of this review is that the PHNEN literature cannot be interpreted reliably by simply counting published cases. Each reported case must first be evaluated for diagnostic credibility. Two separate questions must be answered: first, is the lesion truly a neuroendocrine neoplasm; second, is there sufficient evidence to support a primary hepatic origin rather than metastatic disease?

In addition, each case must be classified according to current WHO morphology‐first criteria. This requires assessment of differentiation, architecture, cytology, necrosis, Ki‐67 and/or mitotic count, immunohistochemical profile, and, when available, molecular features. Because many published reports provide incomplete or inconsistent information, expert pathology review is essential to identify definite, probable, borderline, and insufficiently documented cases.

### 1.5 Objectives

This systematic review had five objectives:

1. To identify published reports of likely PHNEN through a PRISMA‐based systematic search and conventional screening by independent reviewers.
2. To perform second‐level expert pathology adjudication of potentially eligible articles using a predefined diagnostic checklist.
3. To reinterpret sufficiently documented cases according to the WHO 6th edition morphology‐first framework into NET G1, NET G2, NET G3, NEC, MiNEN, amphicrine carcinoma, or paraganglioma.
4. To analyse clinical, immunohistochemical, molecular, and therapeutic data separately for each category.
5. To derive category‐specific diagnostic and management implications, rather than comparative treatment recommendations.

## 2 Methods

### 2.1 Protocol and Registration

This systematic review was conducted in accordance with the PRISMA 2020 guidelines [17] (Figure 1). The review used a two‐step process comprising conventional systematic‐review screening followed by expert pathology‐based reappraisal. The second step was designed specifically to address diagnostic misclassification, which represents a major source of bias in the PHNEN literature.

**FIGURE 1:**
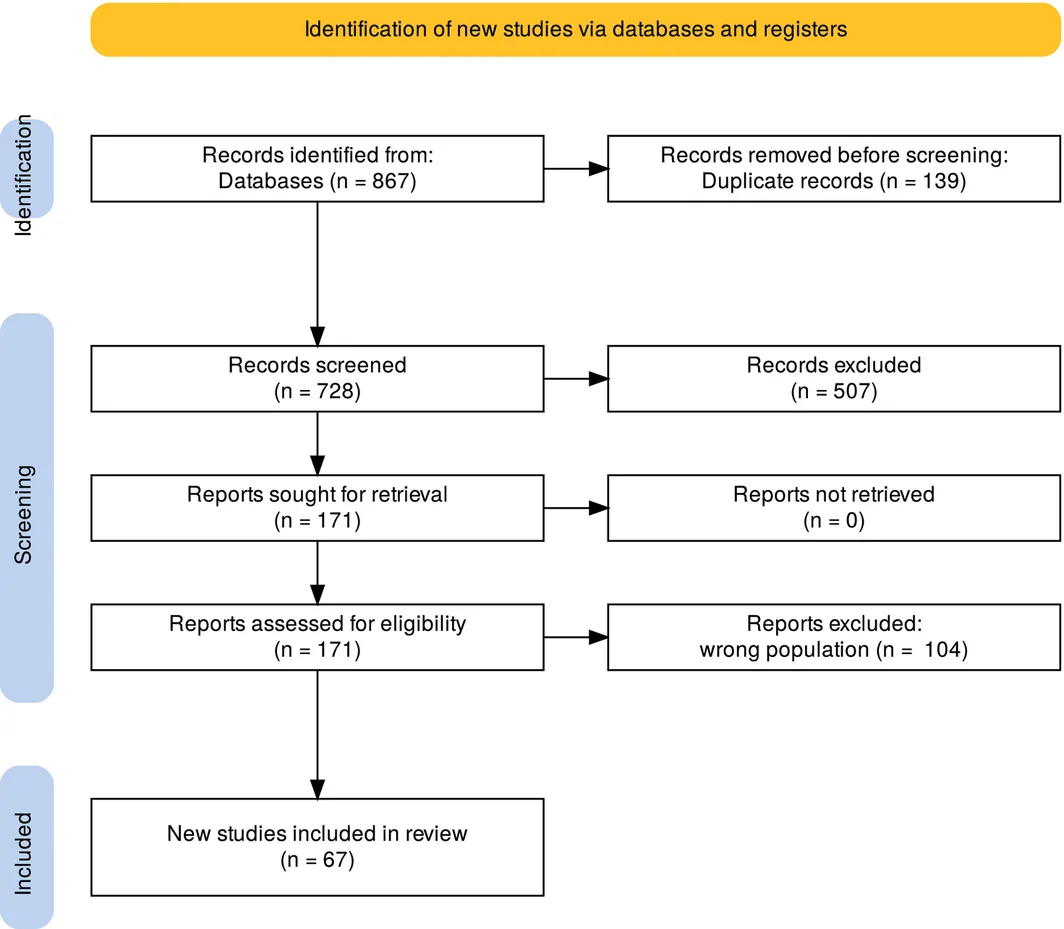
PRISMA flow diagram.

### 2.2 Search Strategy

A systematic literature search was conducted in PubMed/MEDLINE, Scopus, and Web of Science, covering the 15‐year period from January 2010 to December 2025. The search strategy combined keywords and related terms pertaining to primary hepatic neuroendocrine neoplasms, including: ‘primary hepatic neuroendocrine tumour,’ ‘primary hepatic neuroendocrine neoplasm,’ ‘primary hepatic neuroendocrine carcinoma,’ ‘hepatic neuroendocrine tumour,’ ‘hepatic carcinoid,’ ‘liver neuroendocrine tumour,’ ‘primary liver neuroendocrine carcinoma,’ ‘MiNEN liver,’ ‘MANEC liver,’ ‘mixed neuroendocrine–non‐neuroendocrine neoplasm liver,’ ‘amphicrine liver,’ ‘amphicrine‐like carcinoma liver,’ ‘hepatic amphicrine carcinoma,’ and ‘hepatic paraganglioma.’ Given the substantial revision of the classification of neuroendocrine neoplasms introduced by the 2019 World Health Organization classification, studies published from 2019 onward were considered.

### 2.3 Eligibility Criteria

Studies reporting primary hepatic neuroendocrine neoplasms or mixed/amphicrine neoplasms were considered for expert pathology review. Eligible publication types included case reports, case series, retrospective cohort studies, registry studies, and systematic reviews providing extractable individual patient data after case‐level de‐duplication. Eligibility required convincing neuroendocrine morphology, positivity for at least two neuroendocrine markers, compatibility with primary hepatic origin, an adequate work‐up excluding an extrahepatic primary, and extractable clinical or pathological data. Borderline or probable cases were adjudicated separately by expert consensus. The complete eligibility and adjudication pathway is summarised in Figure 2.

**FIGURE 2:**
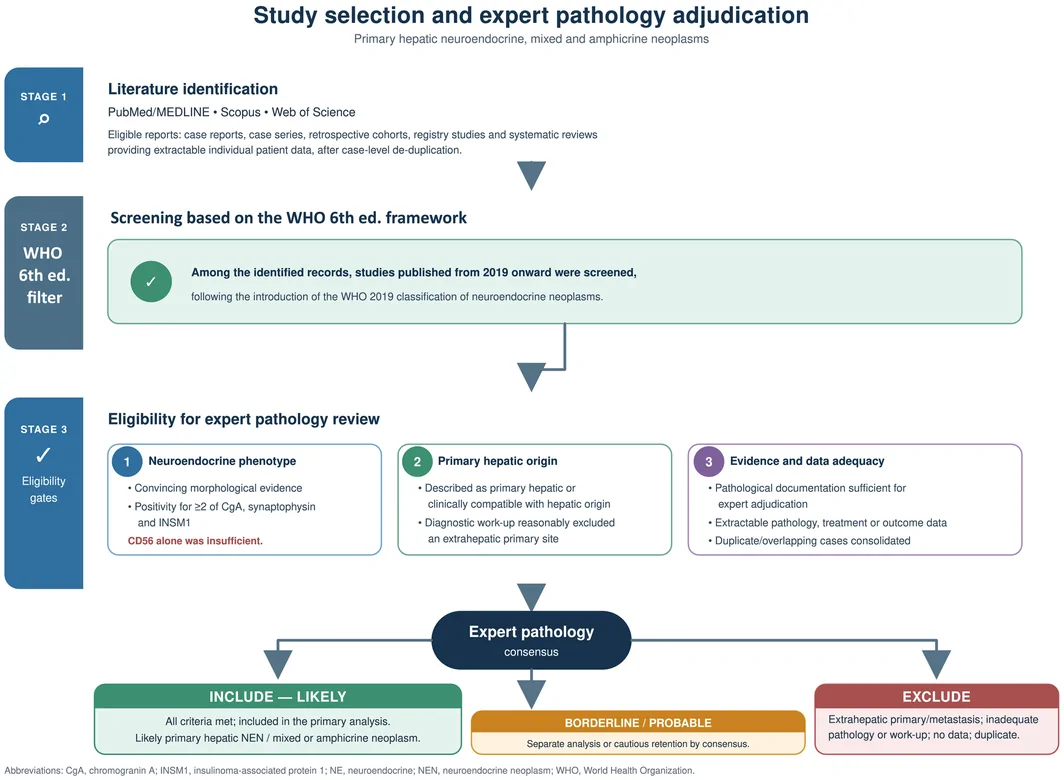
Overview of study development, pathology‐based review and final classification of PHNEN.

### 2.4 Diagnostic Classification: WHO 6th Edition Morphology‐First Approach and Expert Pathology Adjudication

Each paper was reclassified according to the WHO 6th edition morphology‐first framework, with reference to the preceding 2019 and 2022 classifications and the refinements introduced in the recently published 6th edition [9, 10, 12] (Table 2).

**TABLE 2:** WHO 6th edition morphology‐first classification criteria applied to suspected hepatic neuroendocrine neoplasms.

Category	Morphology	Ki‐67	Definition	Necrosis
NET G1	Well‐differentiated	< 3%	Organoid/trabecular architecture, salt‐and‐pepper chromatin	Present/absent
NET G2	Well‐differentiated	3%–20%	Same as G1 with proliferation index within the specified cut‐offs	Present/absent
NET G3	Well‐differentiated	> 20%	Introduced WHO 2017 (pancreas)/2019 (GI); NOT equivalent to NEC	Present/absent
NEC	Poorly differentiated	Variable (typ. > 55%)	Small‐cell or large‐cell poorly differentiated morphology is required; abnormal p53/Rb expression or TP53/RB1‐pathway alterations are supportive but not defining	Variable
MiNEN	Mixed	Variable	Two spatially and morphologically distinct, immunophenotypically confirmed components, each representing at least 30% (e.g., HCC‐NEC)	Present/absent
Amphicrine‐like carcinoma	Single dual‐phenotype tumour‐cell population	Variable	The same tumour cells show simultaneous neuroendocrine and non‐neuroendocrine differentiation; separate components are absent	Present/absent
Paraganglioma	Zellballen	Low	Non‐epithelial NENs, CK‐negative, GATA3‐positive	—

All potentially eligible full‐text articles underwent second‐level pathology adjudication by a panel of expert pathologists with specific expertise in hepatobiliary and neuroendocrine pathology. Each article was reviewed using a predefined diagnostic checklist designed to evaluate both the reliability of the neuroendocrine diagnosis and the plausibility of primary hepatic origin. Each article was reviewed independently by two expert pathologists, and uncertain or discordant cases were discussed by the panel until consensus was reached (Table 3).

**TABLE 3:** Proposed diagnostic and classification algorithm for epithelial PHNENs.

Section	Criterion	Details/Requirements
General criteria	Morphological and immunophenotypic confirmation of a hepatic NEN	Published morphology must be compatible with NET, NEC, MiNEN, or amphicrine‐like carcinoma involving the liver.
	Exclusion of another primary site	No extrahepatic primary is identified by the documented clinical, endoscopic, radiological, and nuclear‐medicine evaluation; this supports but does not prove hepatic origin.
Histology	1. Tumour morphology	Morphology must first establish neuroendocrine differentiation and then support classification as NET, NEC, MiNEN, or amphicrine‐like carcinoma.
	2a. Keratin expression	Keratin expression supports an epithelial NEN and helps separate it from paraganglioma and selected mimics.
	2b. Neuroendocrine markers	At least two of chromogranin A, synaptophysin, and INSM1 should be positive before neuroendocrine differentiation is considered definitively established.
		CD56 may be recorded but does not count towards the two‐marker requirement; limited or focal staining should prompt careful exclusion of mimics.
	3. MiNEN versus amphicrine‐like carcinoma	MiNEN requires two morphologically distinct, immunophenotypically confirmed components, each representing at least 30%. Amphicrine‐like carcinoma requires one tumour‐cell population with simultaneous neuroendocrine and non‐neuroendocrine differentiation.
	4. NET G3 versus NEC distinction	NET G3 should be distinguished from NEC primarily by well‐differentiated versus poorly differentiated morphology.
		NETs require quantitative mitotic and Ki‐67 assessment for WHO grading; tumour necrosis should be reported.
		NECs should be subclassified as small‐cell or large‐cell carcinomas. Abnormal p53/Rb expression or TP53/RB1‐pathway alterations may provide supportive evidence when morphology is difficult.
	5. Exclusion of mimics	Hepatocellular carcinoma, cholangiocarcinoma, metastatic NEN, paraganglioma, and other mimics should be excluded with a morphology‐directed immunohistochemical panel.
Radiology/Nuclear medicine	1. Cross‐sectional imaging	CT or MRI of the chest, abdomen, and pelvis to identify extrahepatic tumour deposits or a potential primary site
	2. Somatostatin receptor imaging	DOTATATE PET/CT or PET/MRI to detect tumour deposits outside the liver
	3. Small bowel evaluation	CT or MR enterography should be considered when an occult small bowel primary is suspected
Additional clinical work‐up	1. Endoscopic evaluation	Upper and lower gastrointestinal endoscopy, with endoscopic ultrasonography when indicated, should evaluate occult primary tumours not detected by imaging.
	2. Clinical correlation and follow‐up	Correlation with the subsequent clinical course is essential; atypical findings should prompt re‐evaluation of histology and imaging studies
Final diagnostic classification	Primary hepatic NET/NEC	If neuroendocrine differentiation is confirmed and no extrahepatic primary is identified after comprehensive evaluation, the lesion may be designated a suspected primary hepatic NET/NEC. Long‐term reassessment is required because hepatic origin cannot be established with certainty.

Briefly, adjudication first required published morphology compatible with neuroendocrine differentiation with positivity for at least two of chromogranin A, synaptophysin, and INSM1. CD56 was recorded when available but did not count towards this minimum confirmation algorithm. For mixed lesions, the reviewers distinguished MiNEN, defined by separate neuroendocrine and non‐neuroendocrine components each comprising at least 30%, from amphicrine‐like carcinoma, defined as either individual tumour cells showing concurrent morphological and immunohistochemical evidence of neuroendocrine and non‐neuroendocrine differentiation, or a morphologically uniform neoplastic population comprising distinct cell subsets that express neuroendocrine or non‐neuroendocrine markers, with an additional subset co‐expressing both phenotypes in some cases.

NET G3 and NEC were separated primarily by differentiation and cytomorphology. Ki‐67 and mitotic activity were used for grading, whereas p53/Rb immunohistochemistry and *TP53/RB1*‐pathway alterations were considered supportive when reported. A high proliferation index or a *TP53/RB1*‐pathway abnormality alone was not considered diagnostic unless supported by morphology. Cases that remained unresolved were classified as high‐grade NEN, NET versus NEC unresolved. The final curated dataset included likely PHNENs, while borderline cases were retained only when expert consensus considered the available clinicopathological evidence sufficiently convincing (as no pathology test establishes hepatic origin unequivocally).

## 3 Results

### 3.1 Study Selection and Category Distribution

After conventional full‐text screening, 67 articles were considered potentially eligible and were submitted to expert pathology adjudication (Figure 1).

### 3.2 Expert Pathology Adjudication and Diagnostic Reliability

On case‐by‐case review, all 28 retained publications documented morphology compatible with neuroendocrine differentiation and positivity for at least two WHO‐recommended general neuroendocrine markers. The most frequent combination was synaptophysin and chromogranin A; INSM1 was reported mainly in more recent publications. CD56 was not counted towards the two‐marker requirement.

Reasons for exclusions were related to the lack of one or more key diagnostic elements. Common limitations included absent or minimal histopathological description, lack of histological images, missing Ki‐67 or mitotic count, unclear WHO grade, absence of site‐of‐origin immunohistochemical markers, and limited documentation of the search for an extrahepatic primary tumour. In some cases, original terminology was inconsistent with current classification [15, 16, 20, 26, 27, 28, 29]. Tumours described as ‘neuroendocrine carcinoma’ were sometimes more compatible with NET based on morphology and available images. Conversely, true NEC required evidence of poorly differentiated small‐cell or large‐cell morphology rather than high Ki‐67 alone [9, 10, 11, 14, 16]. Several cases historically described as ‘well‐differentiated neuroendocrine carcinoma’ were reinterpreted as NET under current WHO terminology. The expert pathology panel therefore performed two distinct functions: first, it excluded or downgraded reports with insufficient diagnostic support; second, it reclassified retained tumours according to modern morphology‐first criteria. All retained mixed neoplasms were reassessed for MiNEN and amphicrine carcinoma and only 2 MiNENs and 1 amphicrine carcinoma were judged to be convincing [30, 31, 32].

### 3.3 Clinical/Imaging/Follow Up Evidence Supporting Primary Hepatic Origin

The evidence supporting primary hepatic origin varied substantially among studies. Diagnostic confidence was based on the combined assessment of cross‐sectional imaging, gastrointestinal and bronchopulmonary evaluation when indicated, somatostatin‐receptor imaging for well‐differentiated tumours, FDG‐PET for high‐grade neoplasms, surgical or autopsy findings, and longitudinal follow‐up without identification of an extrahepatic primary [5, 23, 33, 34, 35, 36, 37, 38, 39, 40]. The strength of primary‐origin evidence was therefore treated as a graded assessment rather than as a binary variable. Reports relying exclusively on CT or MRI with short follow‐up were considered less robust than reports incorporating endoscopy, dedicated pancreatic or small‐bowel evaluation, functional imaging, surgical exploration, autopsy, or prolonged follow‐up.

### 3.4 Final Expert‐Adjudicated Classification

After expert pathology adjudication, cases were categorized as NET, NEC, MiNEN, paraganglioma, or not otherwise classifiable. Importantly, this classification differed in several cases from the terminology used by the original authors. Of note, paragangliomas were analysed separately because of their non‐epithelial neuroendocrine nature [10], and cases that could not be confidently assigned to a modern WHO category were either excluded or classified as not otherwise classifiable, depending on the level of available evidence.

Following expert pathology adjudication, 28 studies (41.8%) were retained in the final pathology‐adjudicated dataset [2, 5, 18, 20, 23, 26, 27, 30, 31, 32, 33, 34, 39, 41, 42, 43, 44, 45, 46, 47, 48, 49, 50, 51, 52, 53, 54, 55], and 39 were excluded because of insufficient evidence supporting hepatic origin, inadequate pathological documentation, probable metastatic disease, duplicate reporting, or inability to classify the lesion according to current WHO criteria. The 28 retained studies are summarised in Table S1. They comprised 3 multi‐patient series and 25 case reports or small case descriptions, representing 57 reported cases (33 male and 25 female patients; age range: 22–78 years). Well‐differentiated NETs accounted for 45 of 57 category‐classifiable cases (78.8%), poorly differentiated NECs for 5 cases (8.8%—most were small cell morphologically), and MiNENs were 2 cases (3.5%) (Table 4). In 4 cases (7%), lesions were high‐grade G3 NENs but, at case‐level morphology, description was insufficient to distinguish NET from NEC. A representative PHNENs well‐differentiated NET G1 is shown in Figure 3. One case (1.8%) was diagnosed as an amphicrine carcinoma primary to the liver [30].

**TABLE 4:** Clinicopathological data of the cases obtained from the selected references following expert pathology review.

Feature	NET (*n* = 45)	NEC (*n* = 5)
Median age, Median (range)	51 years (22–74)	72 years (42–78)
M/F ratio	22/23 (0.96)	5/0 (5.0)
Asymptomatic, *n* (%)	12/29 (41)	2/5 (40)
Tumour size, median, cm (range)	10.5 (2–35)	6 (4.2–17.1)
Dead of disease, *n* (%)	7/39 (18)	2/4 (50%)

*Note:* Only confirmed NET (*n* = 45) and NEC (*n* = 5) were considered, whereas MiNEN (*n* = 2), amphicrine carcinoma (*n* = 1), and high grade NEN, NOS (*n* = 4) were not considered in this clinicopathological analysis due to the small sample size.

**FIGURE 3:**
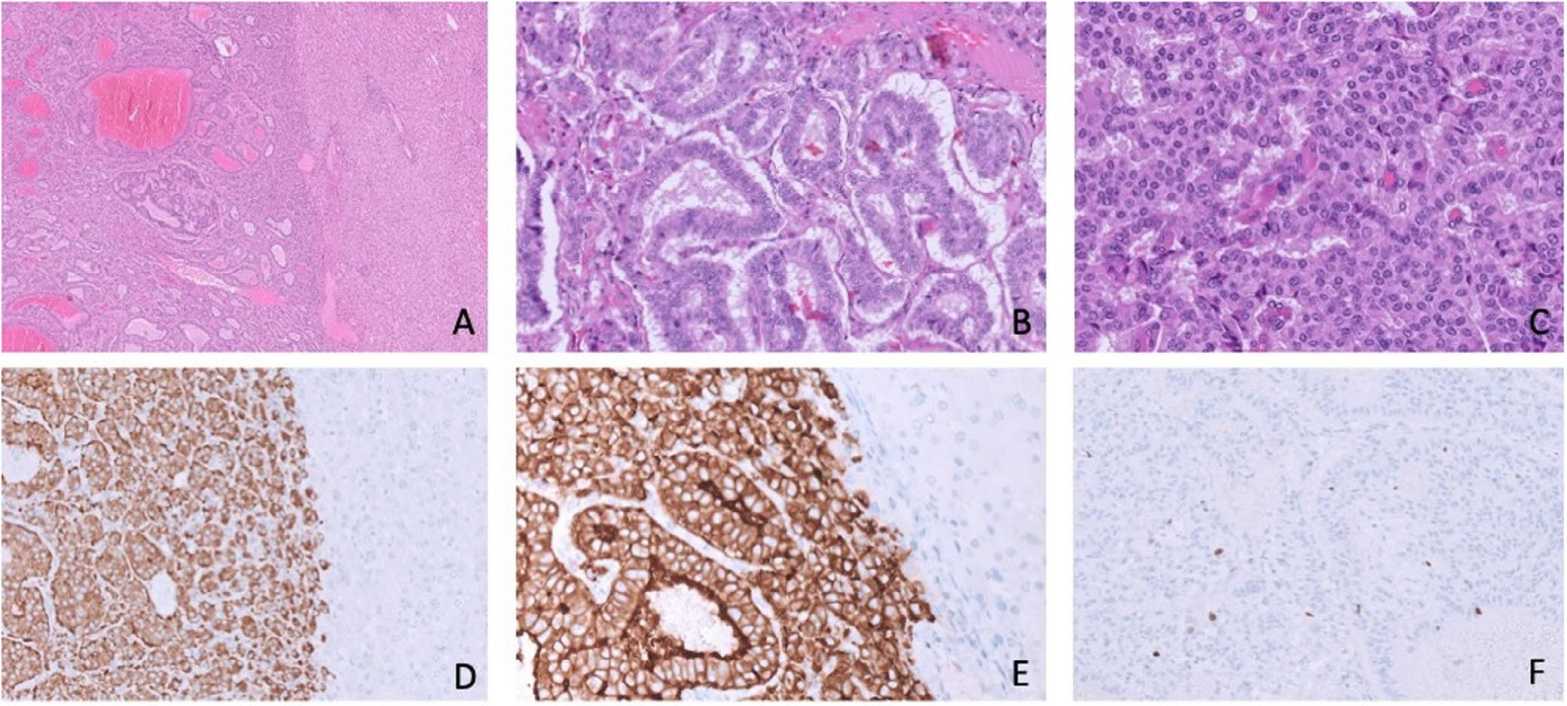
Histopathological and immunophenotypic features of a suspected primary hepatic well‐differentiated neuroendocrine tumour, Grade 1. The patient presented with a solitary 2‐cm nodule in segment 5. 68Ga‐DOTA‐TOC PET showed no extrahepatic sites of disease. Histologically, the tumour was well circumscribed but unencapsulated and showed an intrahepatic organoid proliferation (A), with pseudoglandular (B) and trabecular (C) growth patterns. Tumour showed diffuse and intense synaptophysin expression (D), and intense membranous expression of somatostatin receptor 2A (E). The Ki‐67 proliferation index was 2.5% (F).

Histologic grade was reported in most NETs and NECs (51/54%–94.4%) and distributed as follows: 7 G1 (13.7%), 28 G2 (54.9%), and 16 high‐grade NENs (31.4%), including NET G3 (7 cases), NEC (5 cases), and high‐grade NENs morphology unresolved (4 cases); three cases were ungraded but were well‐differentiated.

Expert‐level revaluation was particularly important in the distinction between NET G3 and NEC and for diagnosis of MiNEN and amphicrine carcinoma.

Hepatic paragangliomas were assessed separately from epithelial NENs. Reported diagnostic features included non‐epithelial neuroendocrine nature, organoid architecture, GATA3, PHOX2B, and tyrosine hydroxylase expression, S100‐positive sustentacular cells, and substantial absence of cytokeratin expression [10]. No hepatic paraganglioma was retained in the final epithelial suspected‐PHNEN dataset.

### 3.5 Category‐Specific Clinicopathological, Treatment, and Outcome Patterns

Because of the predominance of case reports, limited sample sizes, inconsistent follow‐up, and substantial clinical heterogeneity, treatment and outcome findings were summarised descriptively. The work‐up used to exclude an extrahepatic primary varied considerably and was often extremely lacking (Table S1) as some reports relied on cross sectional studies alone [5, 23, 33, 34, 35, 36, 37, 38, 39, 40, 53]. No pooled survival estimates or comparative conclusions regarding treatment effectiveness were considered appropriate as follow‐up duration was inconsistently reported.

#### 3.5.1 NET

Most NET patients presented with a solitary hepatic mass discovered incidentally or during evaluation of nonspecific abdominal symptoms [18, 19, 20, 26, 38, 39, 41, 43, 44, 45, 49, 51, 54, 56, 57, 58, 59, 60]. Right upper quadrant pain, abdominal discomfort, or constitutional symptoms were occasionally reported. Functional syndromes were uncommon and included gastrinoma‐like, insulinoma‐like, or carcinoid‐like presentations in selected cases [21, 23, 53, 61]. Surgical resection was the main treatment for localised disease and was associated with favourable long‐term outcomes in well‐selected cases. Recurrence could occur late, supporting the need for prolonged follow‐up. Liver‐directed therapies, somatostatin analogs, PRRT, and transplantation were reported in selected unresectable or multifocal cases [2, 3, 27, 34, 41, 45, 56, 57, 58, 62]. Because NETs behave differently from NECs, pooling these tumours together with poorly differentiated carcinomas would substantially distort estimates of prognosis and treatment benefit.

NET G3 represents a particularly important category because of the historical risk of misclassification as NEC. NET G3 is defined by well‐differentiated morphology with high proliferative activity, above the G2 range [9, 10, 11, 14, 16], and expert adjudication was especially relevant for this category. Several older reports used terminology that predated the formal recognition of NET G3. In these cases, tumours with preserved well‐differentiated morphology but elevated Ki‐67 may have been reported as NECs. Under the WHO 6th edition framework, these cases should be considered NET G3 rather than NEC when morphology supports well‐differentiated disease [9, 10, 11]. The distinction has direct therapeutic implications. NET G3 may retain somatostatin receptor expression and may be eligible for somatostatin receptor imaging, PRRT, and temozolomide‐based regimens, besides showing poor response to platinum‐based schedules.

#### 3.5.2 NEC

NECs were less frequent than NETs but were associated with more aggressive clinical behaviour. Treatment strategies for NEC differed from those used for NETs. Surgical resection was considered when feasible, but systemic platinum‐based chemotherapy was commonly used for unresectable, metastatic, or rapidly progressive disease [14, 37, 47, 48, 55, 63, 64, 65, 66]. Prognosis remained poor compared with NET.

#### 3.5.3 MiNEN, Amphicrine Carcinoma and Other Rare Entities

MiNENs represented a distinct subgroup of PHNEN containing distinct neuroendocrine and non‐neuroendocrine components, with hepatocellular or cholangiocytic differentiation in the latter [9, 10, 13, 31, 32]. Hepatic MiNENs appeared biologically different from pure NETs. They were more often associated with chronic liver disease, viral hepatitis, cirrhosis, or hepatocellular/biliary differentiation. Treatments of MiNEN were selected according to the reported components [13, 50].

One study reported a single case in a cirrhotic liver of convincing amphicrine carcinoma composed of a single tumour‐cell population with simultaneous hepatocellular (AFP and glypican 3) and neuroendocrine differentiation (synaptophysin and chromogranin) [30].

The inclusion of paraganglioma in PHNEN reviews may be useful for completeness, but these tumours should be analysed separately because their origin, morphology, immunophenotype, and clinical behaviour differ from epithelial PHNENs. They should not be pooled with NETs or NECs in outcome analyses.

## 4 Discussion

The principal finding of this systematic review is that a diagnosis of likely PHNENs should be posed only after detailed pathology‐based evaluation and in‐depth diagnostic work‐up to exclude occult extrahepatic primaries. Indeed, with this in mind, the published literature warrants cautious interpretation as many reports provide incomplete evidence for hepatic origin or insufficient pathological detail for classification under the WHO 6th edition framework.

By combining conventional PRISMA‐based screening with expert pathology review, we generated a curated dataset of cases with stronger clinicopathological support and separated NETs, NECs, MiNENs, paragangliomas, and unresolved non‐classifiable tumours. The pre‐adjudication predominance of publications labelled as NET and the subsequent reclassification show why category‐specific analysis is necessary. This is clinically important because these entities differ substantially in biology, prognosis, imaging phenotype, molecular background, and treatment. Therefore, the traditional practice of pooling all PHNENs into a single category should be abandoned.

Diagnostic validity is the key methodological issue in sPHNEN research. The liver is one of the most common metastatic sites for NENs, and no currently available pathological, radiological, endoscopic, or functional‐imaging test proves hepatic origin. Therefore, the presence of a hepatic neuroendocrine tumour cannot be equated with primary hepatic origin unless extrahepatic primaries have been rigorously excluded.

The WHO 6th edition also strengthens the pathological entry criteria. Neuroendocrine morphology must be present, followed by confirmation with at least two of chromogranin A, synaptophysin, and INSM1; no single marker, and particularly not CD56 alone, is sufficiently sensitive and specific.

These markers establish neuroendocrine differentiation but do not distinguish NET from NEC or establish hepatic origin. The distinction between NET and NEC requires morphological assessment. NET G3 requires well‐differentiated morphology despite proliferation index, whereas NEC requires poorly differentiated small‐cell or large‐cell morphology. TP53/p53 and RB1/Rb abnormalities are useful supportive findings in difficult cases, but morphology remains primary. MiNEN and amphicrine‐like carcinoma must likewise be separated. MiNEN contains two morphologically distinct components, whereas amphicrine carcinomas represent either individual tumour cells showing concurrent morphological and immunohistochemical evidence of neuroendocrine and non‐neuroendocrine differentiation, or a morphologically uniform neoplastic population comprising distinct cell subsets that express neuroendocrine or non‐neuroendocrine markers, with an additional subset co‐expressing both phenotypes in some cases. This distinction is fundamental as it directly affects treatment: NETs may be candidates for surgery, somatostatin receptor imaging, somatostatin analogs, PRRT, CAPTEM, liver‐directed therapies, or transplantation in selected settings, while NECs usually require platinum‐based systemic therapy and have a poorer prognosis. MiNENs require individualised treatment accounting for both components.

Previous reviews have generally prioritised case accumulation. This strategy is understandable given the rarity of PHNENs, but it risks including poorly documented cases or cases classified according to outdated terminology. Maximising sample size at the expense of diagnostic certainty may generate misleading conclusions. For example, including historical NET G3 cases as NEC may overestimate the frequency of NEC and underestimate the prognosis of high‐grade but well‐differentiated tumours. Including metastatic NENs misclassified as primary hepatic tumours may distort survival, recurrence, and treatment‐response estimates. Including MiNENs in pure NET analyses may similarly bias conclusions because MiNENs have different biology and prognosis.

The present review therefore adopted a conservative approach. Rather than maximising case numbers, we prioritised diagnostic validity. This approach may reduce the apparent size of the evidence base, but it provides a more reliable foundation for clinical interpretation as pooled PHNEN survival curves, pooled recurrence rates, or pooled treatment outcomes should be interpreted with extreme caution unless based on expert‐adjudicated category‐specific data.

The diagnosis of PHNEN remains a diagnosis of exclusion. Comprehensive evaluation should include high‐quality liver imaging, whole‐body staging, gastrointestinal endoscopy, colonoscopy, and functional imaging when available. Somatostatin receptor imaging is particularly useful for well‐differentiated NETs, whereas FDG‐PET may be more informative for high‐grade NECs. However, the optimal work‐up may differ according to tumour category. In well‐differentiated NETs.

NETs, somatostatin receptor imaging plays a crucial role in identifying occult tumours. In NEC, FDG‐PET may be more sensitive because poorly differentiated tumours may lose somatostatin receptor expression. Endoscopic ultrasound should be considered when pancreatic origin remains possible. Long‐term follow‐up is also important because an occult extrahepatic primary may become evident only later (possibly years). Future reports should explicitly describe the diagnostic work‐up performed to exclude extrahepatic primary disease. Statements such as ‘no other primary was found’ are insufficient unless accompanied by details of imaging, endoscopy, functional imaging, surgical exploration, autopsy, or follow‐up. Based on the diagnostic limitations identified in the reviewed literature, we propose a simplified stepwise algorithm integrating pathological classification, exclusion of potential mimics, cross‐sectional and functional imaging, endoscopic investigations, and longitudinal reassessment (Figure 4).

**FIGURE 4:**
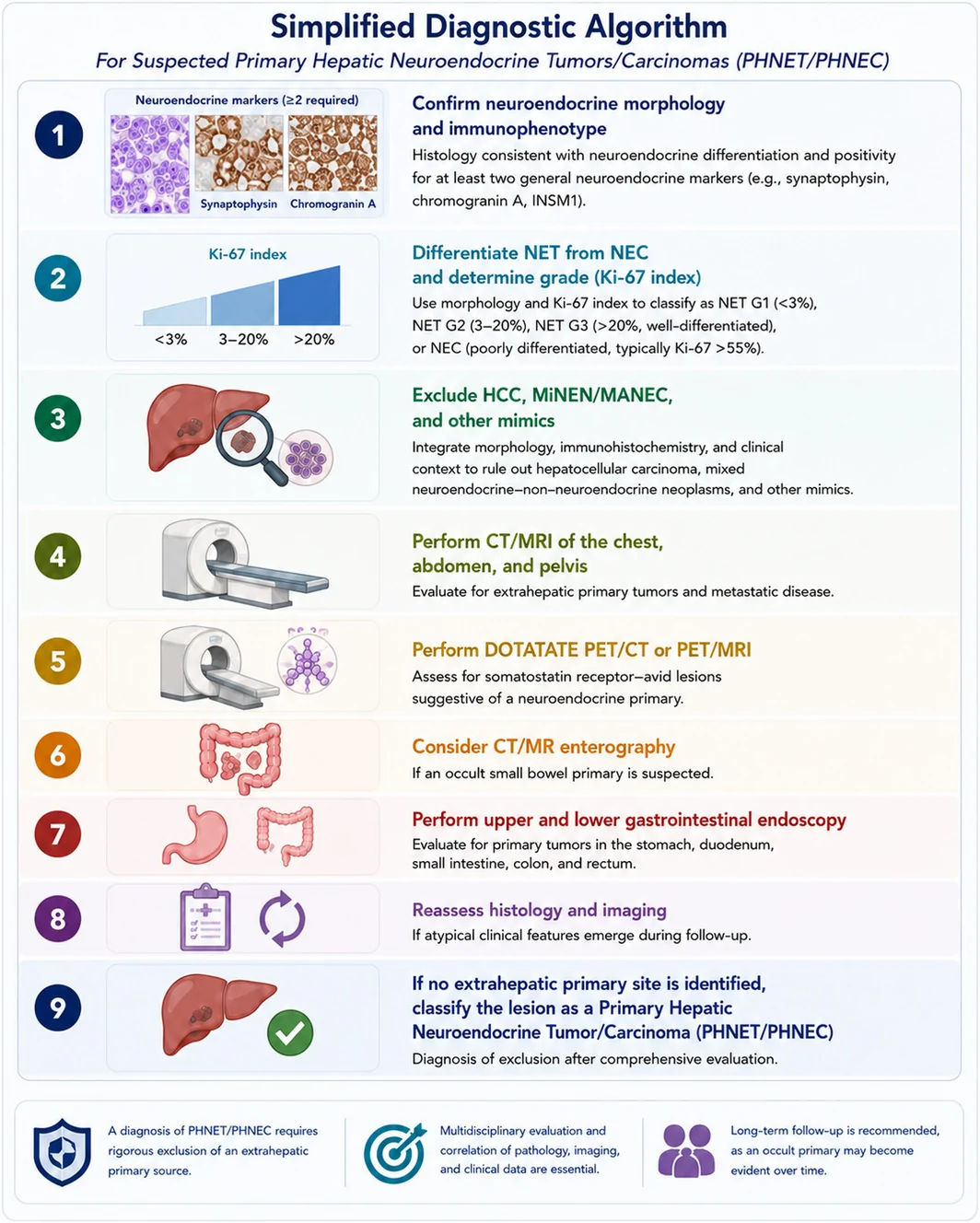
Proposed simplified diagnostic algorithm for suspected primary hepatic neuroendocrine neoplasms; the final designation remains a diagnosis of exclusion after comprehensive evaluation.

The clinical implications of this review are straightforward. First, likely PHNEN should not be considered a single disease. Management should be based on the expert‐adjudicated tumour category. Second, diagnostic confidence matters. A patient with a true primary hepatic NET may be managed very differently from a patient with metastatic small‐bowel NET to the liver. Third, pathology should guide systemic treatment. NET G3 and NEC may both have high Ki‐67, but they are not the same disease and should not automatically receive the same treatment. Fourth, MiNENs should prompt careful evaluation of both components and consideration of liver disease background, including cirrhosis or viral hepatitis. In practical terms, multidisciplinary review involving hepatobiliary surgeons, oncologists, radiologists, nuclear medicine physicians, gastroenterologists, and expert pathologists should be considered mandatory before assigning a diagnosis of sPHNEN.

The main strength of this review is its two‐step methodology. Unlike reviews that primarily aggregate published cases, this study combined systematic literature screening with expert pathology adjudication, including renewed verification of morphology and the WHO 6th edition two‐marker requirement. Some limitations remain. First, expert classifications were based on published descriptions and images rather than central review of original slides or tissue material, and some, therefore, remained probabilistic. Second, the evidence base consisted predominantly of case reports and small retrospective series with heterogeneous reporting, staging investigations, treatment, and follow‐up. Third, publication and selection bias are likely because unusual, surgically treated, or long‐surviving cases may be preferentially reported. Fourth, incomplete case‐level reporting in multi‐patient studies limited category‐specific analyses. Fifth, molecular data relevant to TP53/RB1 pathways were sparse. Sixth, the review cannot provide comparative estimates of treatment effectiveness or prognosis. Finally, even comprehensive negative systemic investigation cannot definitively prove hepatic origin. These limitations justify the term ‘likely’ PHNEN and reinforce the need for international registries incorporating central pathology review, standardised staging, and prospective long‐term follow‐up.

## 5 Conclusions

This systematic review demonstrates that suspected primary hepatic neuroendocrine neoplasms should not be approached as a single homogeneous diagnostic category. The available literature requires critical pathology‐based evaluation and in‐depth exclusion of an extra‐hepatic primary before clinically meaningful conclusions can be drawn. The WHO 6th edition framework requires compatible morphology and at least two recommended general neuroendocrine markers, followed by explicit separation of NET, NEC, MiNEN, amphicrine carcinoma, paraganglioma, and unresolved cases. Future studies and registries should report a minimum diagnostic dataset with standardised terminology according to the WHO 6th edition framework and in‐depth clinical/imaging and follow‐up studies.

## Author Contributions

M.M., O.C., F.G., and A.V. performed study concept and design. M.M. and O.C. performed development of methodology and data acquisition. A.G., F.G., S.L.R., S.U., and A.V. performed critical evaluation of data acquisition. M.M. and O.C. performed writing of the original draft. M.M., O.C., A.G., F.G., S.L.R., S.U. and A.V. performed critical evaluation of the original draft. All authors read and approved the final paper.

## Funding

The authors have nothing to report.

## Ethics Statement

The authors have nothing to report.

## Consent

The authors have nothing to report.

## Conflicts of Interest

The authors declare no conflicts of interest.

## Supporting information


**Table S1:** Characteristics and expert pathology‐based reappraisal of the 28 studies retained in the final sPHNEN dataset.

## Data Availability

All data are provided in the manuscript.
