# Supplementary material for: Primary Hepatic Neuroendocrine Neoplasms: Systematic Review and Expert Pathologist Reappraisal Using the WHO 6th Edition Framework
Source: Liver Int. 2026 Oct 13;46(11):e70921. doi: 10.1111/liv.70921 (PMC13662408; doi:10.1111/liv.70921)
Supplement: Supplementary file 1 [file LIV-46-0-s001.docx]

**Primary Hepatic Neuroendocrine Neoplasms: Systematic Review and Expert Pathologist Reappraisal Using the WHO 6^th^ Edition Framework**

Marcello Maestri^1,2,°^, Ottavia Cicerone^1,2,°^,Alessandro Gambella^3,4,*^, Federica Grillo^3,4,*^, Stefano La Rosa^5^, Silvia Uccella^6,7^, Alessandro Vanoli^8^

**Supplementary Material**

**Supplementary Table 1. Characteristics and expert pathology-based reappraisal of the 28 studies retained in the final sPHNEN dataset**

| **Ref.** | **Study** | **Design / cases** | **Expert classification** | **Main adjudication note** |
| --- | --- | --- | --- | --- |
| 2 | Shah et al., 2019 | Single-center retrospective case series | The series included more than one WHO category and therefore required case-level reassessment. Four cases were retained, including: NET G2 (n=2), NET NOS (n=1), and NEC (n=1) | The clinical work-up included morphological and functional imaging, but the published pathological data were not uniform across patients. |
| 5 | de Mestier et al., 2022 | Molecular series, 16 cases | NET G1 (3 cases);  NET G2 (13 cases) | The study provided detailed pathological and molecular evidence supporting a predominantly well-differentiated NET G2 cohort. |
| 18 | Hu et al., 2019 | Case report | NET G2 | The report documented multiple hepatic lesions, but the investigation for an occult extrahepatic primary was limited; hepatic origin was therefore considered probable rather than certain. |
| 20 | Lv et al., 2025 | Case report | NET, probably G2 | The historical small-cell terminology was not supported by the published well-differentiated morphology, and somatostatin-receptor imaging was not reported. |
| 23 | Kriger et al., 2020 | Case report | Functional NET G2, insulinoma | Detailed pathological findings and the clinical investigation supported a suspected hepatic insulin-producing NET. |
| 27 | Pastrian et al., 2019 | Two cases | One retained case: NET G3 | One case met criteria for well-differentiated NET G3; the second had unresolved large-cell NEC versus NET morphology and insufficient exclusion of an extrahepatic primary and was not retained. |
| 28 | Jung et al., 2019 | Case series, 13 cases | Case-level reclassification required. Ten cases retained,including  NET G1 (n=3), NET G2 (n=2) High grade NEN NOS (n=4), and NET NOS (n=1). | The clinical series was informative, but published case-level morphology did not consistently permit separation of NET from NEC. |
| 32 | Sakamoto et al., 2021 | Case report | NET G2 | A surgical specimen, confirmatory immunohistochemistry, Ki-67, CT, MRI, and FDG-PET were documented. |
| 35 | Oczkowicz et al., 2019 | Case report | NET G1 | Somatostatin-receptor imaging supported liver-confined disease, although the published morphological description was limited. |
| 36 | Nikeghbalian et al., 2020 | Case report | NET G3 | The report documented Ki-67 and a confirmed neuroendocrine phenotype, but provided only a limited morphological description. |
| 37 | Khan et al., 2021 | Case report | NET G2 | Detailed morphology, confirmatory neuroendocrine immunohistochemistry, site-of-origin markers, and clinical investigation were provided. |
| 38 | Zhang et al., 2022 | Case report | NET G2 | Detailed pathological findings and gallium-68 PET/CT provided comparatively strong support for the diagnosis. |
| 44 | Tang et al., 2023 | Two-case report | NET G2 and NET G3 | CT, MRI, FDG-PET, and somatostatin-receptor imaging were reported, whereas the published pathological detail was incomplete. |
| 45 | Jiang et al., 2024 | Case report | NET G2 | Ki-67 and confirmatory immunohistochemistry were documented, and four-year follow-up strengthened, but did not prove, suspected hepatic origin. |
| 48 | Huang et al., 2020 | Case report | NEC , small cell subtype | Poorly differentiated small-cell morphology, confirmatory neuroendocrine immunohistochemistry, and proliferative activity supported NEC; exclusion of an extrahepatic primary relied mainly on CT. |
| 50 | Kobayashi et al., 2025 | Case report | NEC , small cell subtype | The published poorly differentiated morphology and clinical course supported NEC, although the cytokeratin panel was incomplete. |
| 53 | Cao et al., 2023 | Case report | NEC , small cell subtype | The diagnosis was based on biopsy; CT, MRI, and gastrointestinal endoscopy were performed, but functional imaging was not reported. |
| 58 | Luo et al., 2025 | Case report | NET G3 | Detailed morphology, confirmatory immunohistochemistry, and proliferative assessment supported NET G3; the clinical work-up supported suspected hepatic origin. |
| 59 | Watanabe et al., 2025 | Case report | MiNEN | Morphological, immunophenotypic, and molecular findings supported MiNEN with distinct components; exclusion of an extrahepatic primary relied mainly on cross-sectional imaging. |
| 65 | Zhang et al., 2023 | Case report | NET G3 | Dual-tracer FDG and somatostatin-receptor PET/CT contributed to staging and the search for an extrahepatic primary. |
| 66 | Almas et al., 2020 | Case report | NET, grade not specified | Pathological reporting was limited; negative postoperative gallium PET and follow-up exceeding three years supported, but did not prove, suspected hepatic origin. |
| 68 | Cruz et al., 2025 | Case report | NET G2 | DOTATATE PET/CT, upper endoscopy, and colonoscopy were documented, but histological images were not provided. |
| 69 | Kushwaha et al., 2024 | Case report | Functional NET G2 | The report provided comprehensive pathological findings and clinical investigation supporting a suspected functional hepatic NET. |
| 72 | Wong et al., 2021 | Case report | NET G2, | The pathological findings were compatible with NET G2; PET and 16-month follow-up were reported. |
| 76 | Han et al., 2020 | Case report | NEC, small cell subtype | Biopsy morphology, confirmatory immunohistochemistry, and Ki-67 were reported; exclusion of an extrahepatic primary was limited to CT and six months of follow-up. |
| 78 | Shi et al.,  2021 | Case series 4 cases | Two cases retained: amphicrine carcinoma and NET G3 | Amphicrine carcinoma: biopsy morphology, confirmatory immunohistochemistry, and Ki-67 were reported.  NET G3: surgical resection with detailed morphology and confirmatory immunohistochemistry, and Ki67 |
| 79 | Tsuji et al., 2025 | Case report | MiNEN (HCC+NEC) | Surgical resection with detailed morphology, confirmatory immunohistochemistry, and Ki67 |
| 80 | Tanaka et al., 2022 | Case report | MiNEN (HCC+NEC) | Surgical resection with detailed morphology, confirmatory immunohistochemistry, and Ki67 |

*NET, neuroendocrine tumor; NEC, neuroendocrine carcinoma; MiNEN, mixed neuroendocrine–non-neuroendocrine neoplasm; IHC, immunohistochemistry; SSTR, somatostatin receptor; FDG-PET, fluorodeoxyglucose positron emission tomography. All retained publications documented morphology compatible with neuroendocrine differentiation and positivity for at least two of chromogranin A, synaptophysin, and INSM1; CD56 was not counted toward this threshold. Expert classifications were based exclusively on the pathological and clinical information available in the published reports and do not represent central review of original histological material. The term suspected PHNEN is used because current investigations cannot establish hepatic origin with certainty. In multi-case publications, only individually eligible and sufficiently documented cases were included in category-specific analyses.*
